# Flexible and Self-Healing Aqueous Supercapacitors for Low Temperature Applications: Polyampholyte Gel Electrolytes with Biochar Electrodes

**DOI:** 10.1038/s41598-017-01873-3

**Published:** 2017-05-10

**Authors:** Xinda Li, Li Liu, Xianzong Wang, Yong Sik Ok, Janet A. W. Elliott, Scott X. Chang, Hyun-Joong Chung

**Affiliations:** 1 Department of Chemical and Materials Engineering, University of Alberta, Edmonton, Alberta, T6G 1H9 Canada; 2 School of Natural Resources and Environmental Science & Korea Biochar Research Center, Kangwon National University, Chuncheon, 24341 Korea; 3 Department of Renewable Resources, University of Alberta, Edmonton, Alberta T6G 2H1 Canada

## Abstract

A flexible and self-healing supercapacitor with high energy density in low temperature operation was fabricated using a combination of biochar-based composite electrodes and a polyampholyte hydrogel electrolyte. Polyampholytes, a novel class of tough hydrogel, provide self-healing ability and mechanical flexibility, as well as low temperature operation for the aqueous electrolyte. Biochar is a carbon material produced from the low-temperature pyrolysis of biological wastes; the incorporation of reduced graphene oxide conferred mechanical integrity and electrical conductivity and hence the electrodes are called biochar-reduced-graphene-oxide (BC-RGO) electrodes. The fabricated supercapacitor showed high energy density of 30 Wh/kg with ~90% capacitance retention after 5000 charge–discharge cycles at room temperature at a power density of 50 W/kg. At −30 °C, the supercapacitor exhibited an energy density of 10.5 Wh/kg at a power density of 500 W/kg. The mechanism of the low-temperature performance excellence is likely to be associated with the concept of non-freezable water near the hydrophilic polymer chains, which can motivate future researches on the phase behaviour of water near polyampholyte chains. We conclude that the combination of the BC-RGO electrode and the polyampholyte hydrogel electrolyte is promising for supercapacitors for flexible electronics and for low temperature environments.

## Introduction

Enhancing the low temperature performance of electrochemical storage devices is crucial in applications in automobiles, wearable devices, and energy grids in cold climates. At low temperatures (<−10 °C) such devices suffer various issues, including reduced ion transport due to increased viscosity and embrittlement of polymeric binder components^1^. For electrochemical capacitors, *i*.*e*., supercapacitors, low temperature operation is established down to −40 °C, typically by using organic solvents or ionic liquids with low freezing points^2–4^. However, these liquids have other limitations, such as humidity dependent conductivity change, toxicity and environmental contamination when leaked, as well as high flammability and high vapor pressure, which may lead to hazardous explosions if local overheating occurs^5–9^. To mitigate potential hazards, active research is ongoing on aqueous electrolytes for energy storage devices, such as lithium ion^10^ or sodium ion batteries^11^, as well as supercapacitors^12^ in order to develop safe energy storage devices, but low temperature operation for aqueous electrolytes has not been established. There is a critical need to develop electrochemical energy storage devices that have desirable performance characteristics at low temperatures, with both suitable electrolyte and electrode.

Gel polymer electrolytes, swollen polymers containing electrolyte solutions with proper solvents or plasticizers, possess superior properties such as self-supporting shape and fast ionic transport, properties possessed by solid and liquid electrolytes, respectively. Compared to liquid electrolytes, gel polymer electrolytes are intrinsically free of leakage problems or the need for separators, reducing the requirement for costly special packaging^13^. Poly(vinyl alcohol) (PVA) is one of the widely used polymeric frameworks for aqueous gel polymer electrolytes with proton^14, 15^ or alkaline doping^16, 17^. Potassium hydroxide (KOH) doped PVA hydrogels exhibit high ionic conductivities. However, the low temperature application of aqueous gel electrolytes is limited due to monolithic ice formation. Hydrogels from polyampholytes—polyelectrolytes containing both anion and cation groups in a single chain—possess desirable structural properties found in gel polymer electrolytes such as rubber-like elasticity, extreme tear resistance against crack propagation, self-healing ability and self-adjusting adhesion^18–20^. In addition, our parallel research showed that polyampholyte hydrogel electrolyte maintains mechanical flexibility at very low temperature by slush-like ice formation^21^; thus we envision making robust, flexible, and eco-friendly aqueous energy storage devices suitable for cold climates.

Carbon-based materials have been of intense interest as electrodes for making energy storage devices. While activated carbon from coconut shell is the current gold standard in the supercapacitor industry^22^, carbon nanotubes and graphene have been extensively studied^23, 24^. While most research focuses on nanostructure optimization for maximizing capacitance density by efficient ion transport and sieving, the key issue for industrial application lies in the optimal balance between material production cost and performance. In addition, low volumetric energy density caused by ‘fluffiness’ impedes the use of alternative carbon sources for making energy storage devices^22^.

Biochar (BC) produced from agriculture waste by slow pyrolysis at low temperature (400~700 °C) has attracted attention for soil fertility improvement, carbon sequestration and water purification^25, 26^. Recent studies show possibilities of using biochar in energy storage devices^27–29^. Pure BC is not suitable as an electrode material for three reasons: low specific capacitance, the powdery nature of the material, and low electrical conductivity. Nitric acid treatment and thermal flashing can increase the specific capacitance of exfoliated biochar electrodes from 2.1 to 221.3 F g^−1^ ^28^. Structural integrity and electrical conductivity are remedied by adding polymeric binders and conductive additives, respectively. These additional components make up a considerable fraction of the weight of electrodes, resulting in lower energy density and higher device cost^30–32^.

In this paper, we report the fabrication of a flexible and self-healing aqueous supercapacitor for low temperature applications using polyampholyte and biochar as base materials for gel electrolyte and electrode, respectively. Specifically, we employed a polyampholyte hydrogel as an electrolyte for electrochemical energy storage for the first time due to its preferable mechanical properties such as stretchability, tear-resistance, adjustable adhesion, and self-healing ability. More importantly, we found that polyampholyte hydrogels (PA) prohibited monolithic ice formation; this leads to device flexibility in ice-forming temperatures, which will enable low temperature application of aqueous electrolytes as a cost-effective and eco-friendly solution for use in cold climates. For electrodes, we employed a soybean stover-based biochar with 7.5% (wt) reduced graphene oxide (BC-RGO) as a novel high-performance and cost-effective material. Using KOH to provide electrolytic ions, the polyampholyte-based supercapacitor (SC-PA) achieved specific capacitance of 193 F g^−1^ at 0.5 A g^−1^ with an energy density of 30 Wh kg^−1^ at room temperature. At −30 °C, its energy density was 10.5 Wh kg^−1^, which is remarkably higher than that of a control sample (3.4 Wh kg^−1^) consisting of BC-RGO electrodes with an unconfined KOH solution in a cellulose separator as the electrolyte.

## Results and Discussion

### Device Fabrication: an Overview

**Figure 1:**
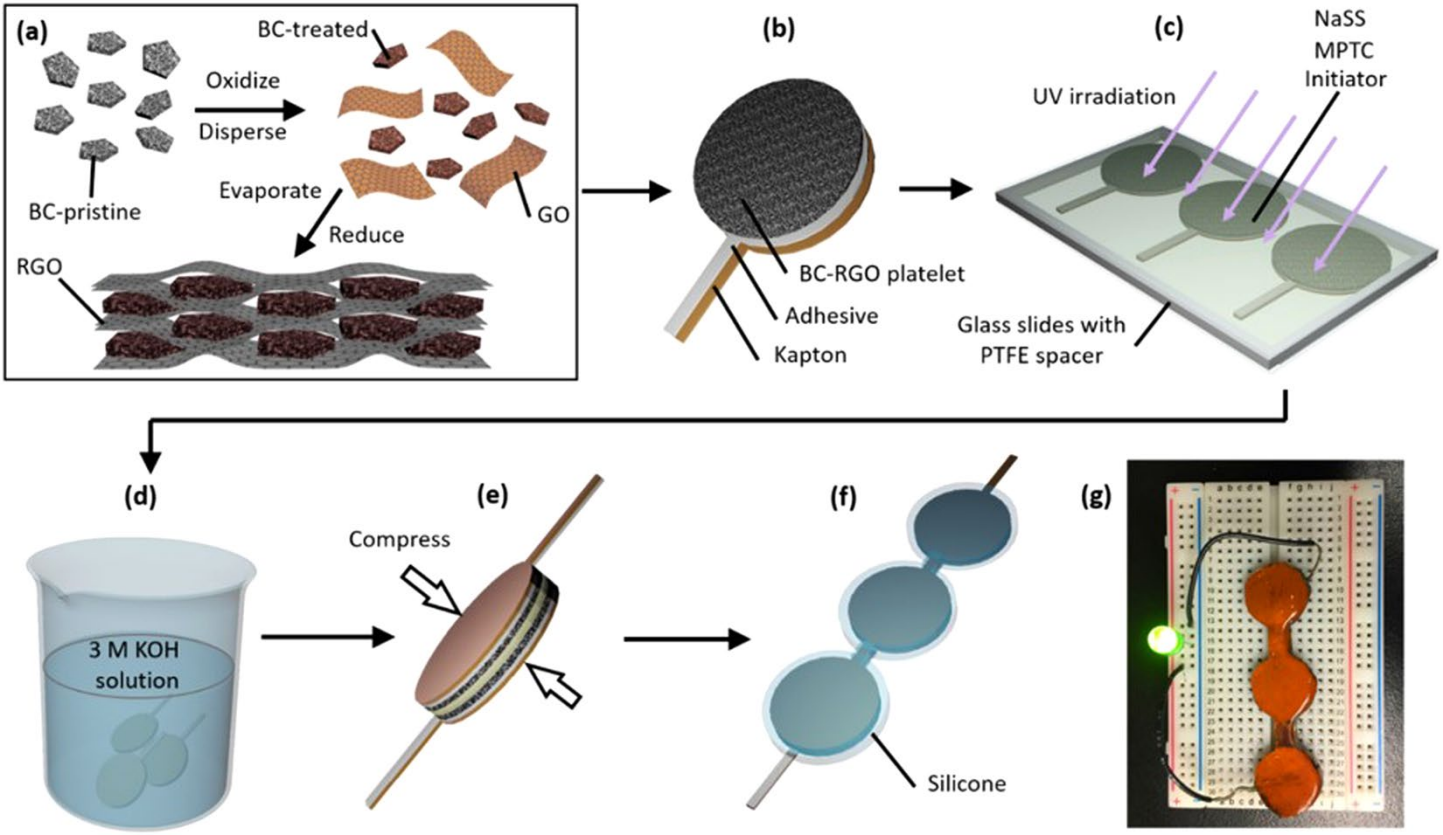
Schematic of supercapacitor (SC-PA) fabrication. (**a**) As-received biochar (BC-pristine) was oxidized (BC-treated) and dispersed in a graphene oxide solution. Subsequent solvent evaporation and graphene oxide reduction result in a consolidated electrode (BC-RGO) with high electrical conductivity. (**b**) The BC-RGO electrodes are supported on a Kapton substrate. (**c**) A polyampholyte hydrogel is synthesized on the BC-RGO electrodes by photo-initiated random copolymerization of NaSS and MPTC. (**d**) The electrolyte is dialyzed in 3 M KOH solution. (**e**) Compressing the dialyzed electrolyte/electrode pair with the top BC-RGO electrode will make a symmetric supercapacitor. (**f**) Three symmetric supercapacitors are encapsulated in silicone to light a green LED in (**g**).

Figure 1 summarizes the procedure for SC-PA fabrication. Pristine biochar processed from soybean stover (BC-pristine) is ground, sieved and acid treated (Fig. 1a). The treated biochar (BC-treated), however, is still in a powder form and is not electrically conductive. The addition of reduced graphene oxide (RGO) (7.5 wt%) provided a binder to maintain mechanical integrity and a conducting conduit for electrical conductivity. The composite is bendable and stretchable without powder disintegration, which is a suitable trait for making flexible devices. After bonding the composite electrode to a Kapton substrate (Fig. 1b), copolymerization of sodium 4-vinylbenzenesulfonate (NaSS) and [3-(methacryloylamino)propyl]trimethyl-ammoniumchloride (MPTC) on the BC-RGO film in a UV chamber synthesizes the hydrogel of poly(NaSS-co-MPTC), denoted as PA (Fig. 1c). Subsequently, the BC-RGO/PA pair is dialyzed in a 3 M KOH solution for 1 day (Fig. 1d). A symmetric supercapacitor is assembled by pressing together two BC-RGO electrodes without the use of a separator (Fig. 1e). Figure 1f describes the encapsulation process, which concludes the fabrication of the supercapacitor (SC-PA). A photograph of a SC-PA in operation is shown in Fig. 1g.

### Electrode: Morphological Characterization

**Figure 2:**
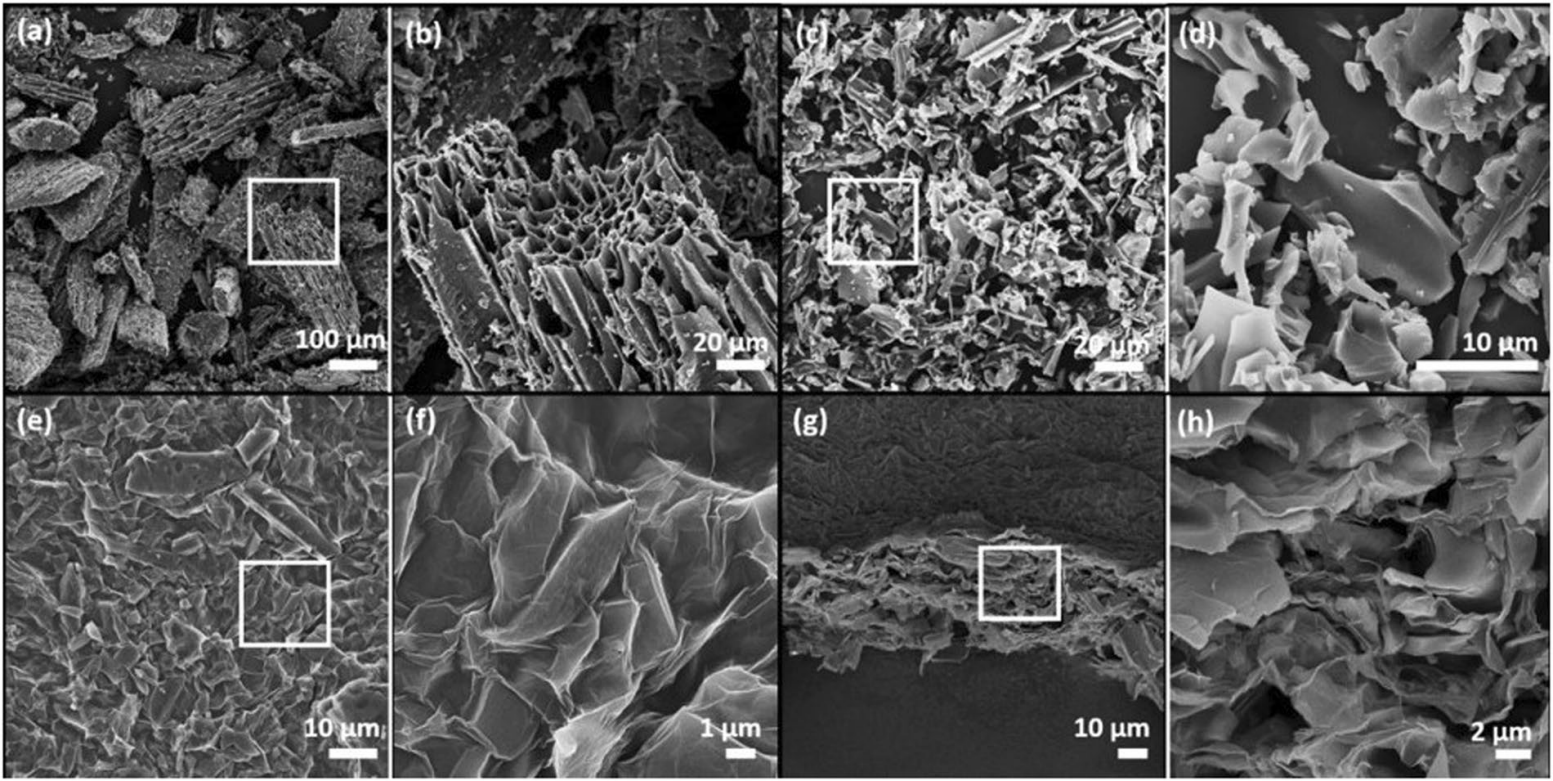
(**a**) The SEM image of as-received biochar (BC-pristine) and (**b**) a magnified image of the small region identified by the white rectangular box in (**a**). (**c**) The SEM image of oxidized biochar (BC-treated) and (**d**) a magnified image of the small region identified in (**c**). (**e**) The SEM image of the top of a BC-RGO electrode and (**f**) a magnified image of the small region identified in (**e**). (**g**) The SEM image of the BC-RGO cross section and (**h**) a magnified image of the small region identified in (**g**).

The morphological evolution of BC was examined by scanning electron microscopy (SEM) and transmission electron microscopy (TEM). The SEM images in Fig. 2a–d show that the milling and the acid treatment reduced the average powder size of BC from ~100 μm to ~10 μm. The TEM images show that the treatment generated an abundance of ~10 nm sized mesopores in BC-treated (Figure S2c), which is not found in BC-pristine (Figure S2a), with micropores and nanopores that are present all over the BC-treated (Figures S2d). The hierarchical porous structure is a clear indication of increased specific surface area. The top-view of the BC-RGO film (Fig. 2e and f) highlights that the BC particles were well wrapped by RGO, resulting in structural integrity. The continuous RGO networks also provide electrical conductivity for the electrode. The cross-sectional views of BC-RGO (Fig. 2g and h) indicate that the thickness of the electrode is ~40 µm. The BC-RGO contains macropores that can serve as ionic pathways for effective diffusion of electrolytes^31, 33^.

**Figure 3:**
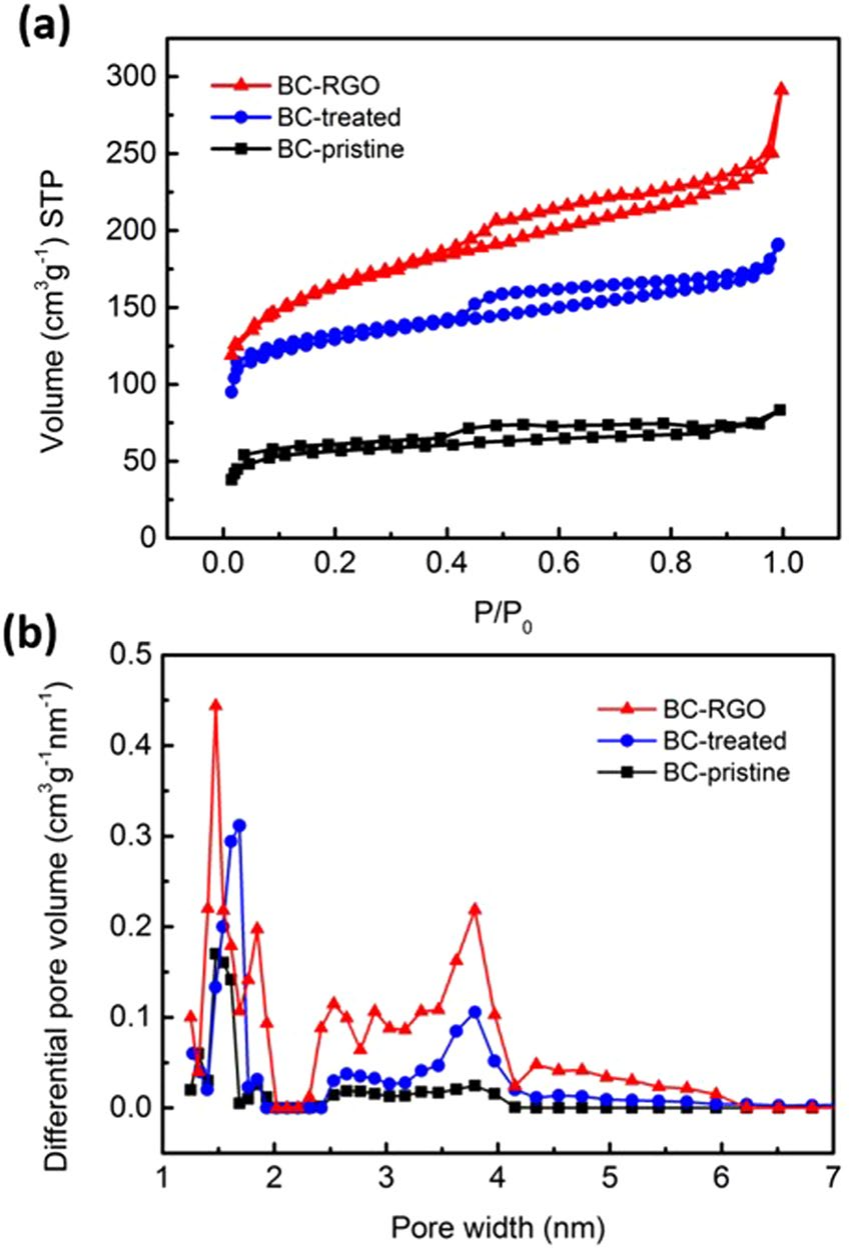
(**a**) Nitrogen adsorption isotherm according to the BET model and (**b**) Pore size distribution (represented as differential pore volume plotted against pore width) derived from (**a**), calculated with the NLDFT model.

The Braunauer–Emmett–Teller (BET) nitrogen adsorption method was used to determine the surface area of BC materials. The adsorption isotherms (volume of nitrogen per gram of BC material at standard temperature and pressure (STP); Fig. 3a) reveal that the specific surface areas of BC-pristine and BC-treated were 187 and 414 m^2 ^g^−1^, respectively, which is consistent with TEM results (Figure S2) and earlier studies on acid treated BC^28, 34^. The RGO wrapping further increased the specific surface area to 483 m^2 ^g^−1^. The pore size distribution of the microspores and mesopores were evaluated by Non-Local Density Functional Theory (NLDFT) (an indicator of pore size distribution; Fig. 3b). For all three samples, there exists a sharp peak at 1.5 nm and a broad peak ranging from 2 to 4 nm, and the peak heights increase from BC-pristine to BC-treated, then to BC-RGO. It is notable that nanometer-scale mesopores (TEM image; Figure S2c) and macropores (Fig. 2h) are also abundant in BC-RGO, indicating a hierarchical porous structure suitable for SC electrodes^35^.

### Electrochemical Test of BC-RGO Electrodes

The electrochemical properties of BC-RGO electrodes were evaluated using three electrodes with Ag/AgCl as a reference electrode. The results of cyclic voltammetry (CV) profiles at different scan rates, galvanostatic charging/discharging (GCD) at different current densities, electrochemical impedance spectroscopy (EIS), and CV performance in various aqueous electrolytes are shown in Figure S4. In all cases, no obvious redox peaks were observed. The general characteristics of CV plots were similar to prior studies on pyrolyzed biomass in KOH (Figure S4a)^36–38^. The symmetric triangle shape of the GCD profiles implies reversibility and stability of electrode materials (Figure S4b). At a current density of 0.5 A g^−1^, the specific capacitance of BC-RGO electrodes reached 216 F g^−1^ (183 F cm^−3^). The capacitance retention was 76% at 6 A g^−1^. The specific surface area normalized capacitance was 44.62 µF cm^−2^ at 0.5 A g^−1^. From the Nyquist plot, the equivalent series resistance (ESR) in the high-frequency region was ~0.6 Ω (Figure S4c)^39^. Detailed discussions can be found in the Supplementary Information.

### Performance of SC-PA at Room Temperature

**Figure 4:**
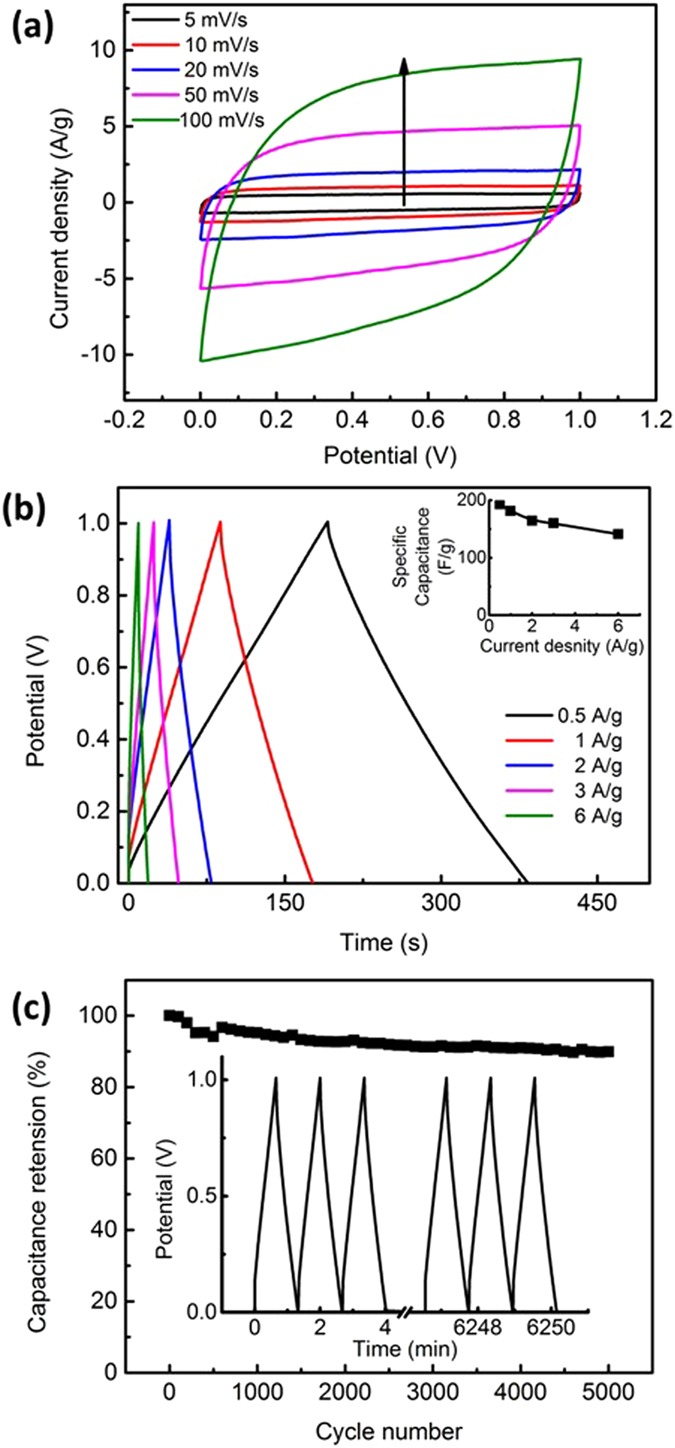
(**a**) Cyclic voltammetry (CV) and (**b**) galvanostatic charging–discharging (GCD) profiles of symmetric SC-PA KOH (inset: specific capacitance verses current density). The arrow in (**a**) indicates the direction of increasing scanning rate. (**c**) Cycle test of the fabricated supercapacitor (SC-PA). The inset presents first and last three galvanostatic charging–discharging (GCD) profiles.

In order to evaluate the efficacy of the polyampholyte hydrogel as a gel electrolyte material, we devised a control sample that employs a liquid aqueous KOH solution with a cellulose separator as an electrolyte (SC-KOH; configuration shown in Figure S5). The polymer, water, and KOH concentrations of control samples were tuned to be same as the polyampholyte-KOH electrolyte, by precisely controlling the amount of KOH solution in the cellulose separator. Figure 4a shows the CV profiles of SC-PA with scan rates ranging from 5 to 100 mV s^−1^. The curves display a quasi-rectangular and symmetric shape. Figure 4b presents the GCD curves of SC-PA at current densities ranging from 0.5 to 6 A g^−1^. At 0.5 and 6 A g^−1^, the specific capacitances of BC-RGO were 193 and 141 F g^−1^, respectively. At a current density of 2 A g^−1^, a capacitance retention of ~90% was obtained after 5000 successive GCD cycles (Fig. 4c).

Energy density and power density are important properties of a supercapacitor. The Ragone plots of SC-PA and SC-KOH are compared with previous studies on biomass-based symmetric supercapacitors with aqueous electrolytes (Figure S7). The SC-PA has energy densities of 30.3, 23.4, and 11.9 Wh kg^−1^ at power densities of 50 W kg^−1^, 1 kW kg^−1^, and 12 kW kg^−1^, respectively. The energy density of SC-PA is comparable to other published supercapacitors that employ coconut shell-based electrodes (state-of-the-art)^40^, as well as other biomass-based electrodes^37, 41^. It should be noted that the activated carbon materials mentioned were produced at 800 °C followed by chemical activation, and the values calculated were on the basis of active components in electrodes; typically, 5–20% of the total electrode mass is binders or conductive additives, which do not exist in our device.

### Performance of the Supercapacitor at Low Temperature

**Figure 5:**
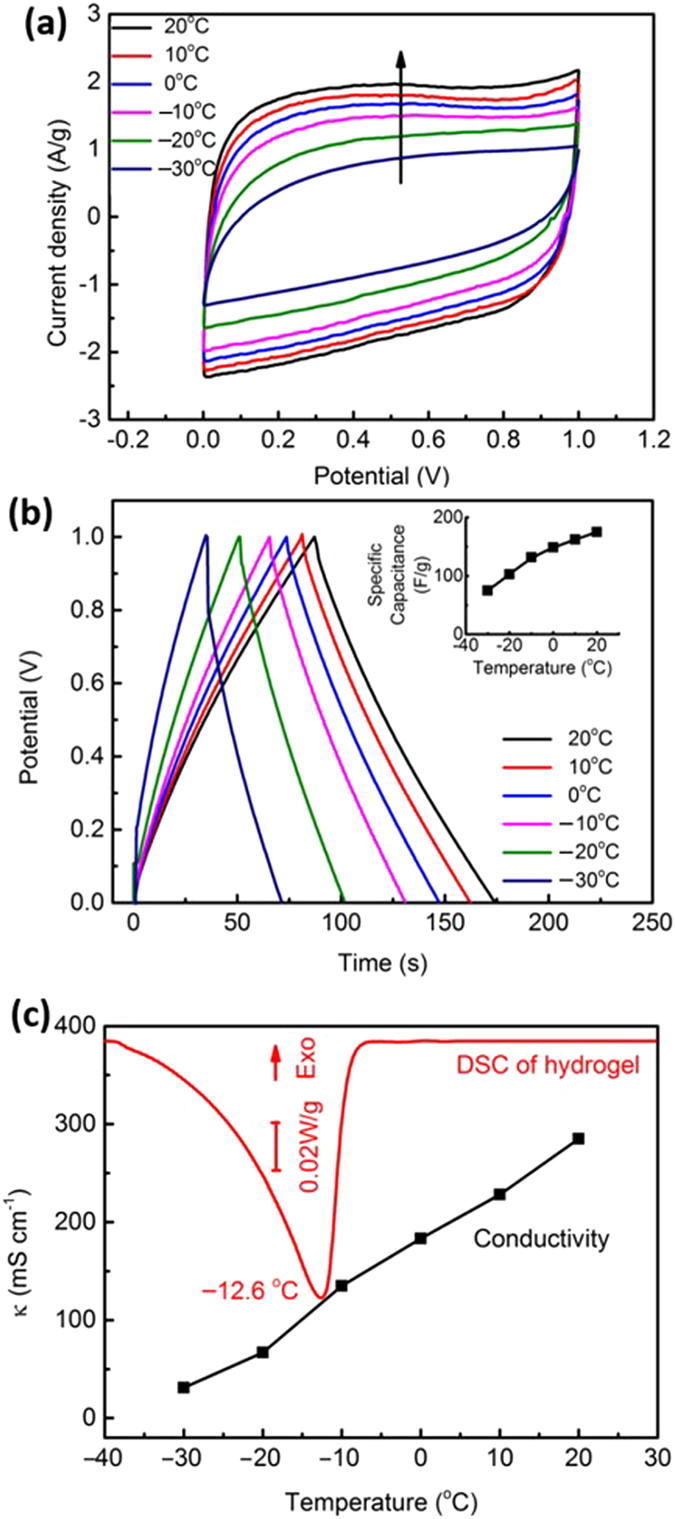
(**a**) Fabricated supercapacitor (SC-PA) temperature dependence of cyclic voltammetry (CV) profiles at a scan rate of 20 mV s^−1^. The arrow indicates the direction of increasing temperatures. (**b**) Galvanostatic charging–discharging (GCD) profiles at a current density of 1 A g^−1^. The inset indicates calculated specific capacitance with respect to temperature change. (**c**) A differential scanning calorimetry (DSC) result of 3 M KOH-containing polyampholyte hydrogel with increasing temperature compared with the change in conductivity as a function of temperature.

The performance of the SC-PA was evaluated at temperatures ranging from 20 to −30 °C. Figure 5a shows the CV profiles of the SC-PA at different temperatures. The sweep rate was fixed at 20 mV s^−1^. With a decreasing temperature, the area enveloped by the CV curve decreased, while the increased resistance to ionic transport caused the shape of the cycle to deviate from a quasi-rectangle. Figure 5b shows the GCD curves of the SC-PA at a charging–discharging current of 1 A g^−1^. Calculated specific capacitance verses temperature is given as an inset in the same figure. The values were 175, 163, 149, 132, 102, and 75 F g^−1^ at temperatures of 20, 10, 0, −10, −20, and −30 °C, respectively. The energy density of the SC-PA was 10.5 Wh kg^−1^ at a power density of 500 W kg^−1^. As indicated by the CV curves shown in the Supplementary Information (Figure S8), the specific capacitance of the control SC-KOH was only 24 F g^−1^ at −30 °C, where the energy density was 3.4 Wh kg^−1^ at a power density of 500 W kg^−1^, which is only 32% of the performance of the SC-PA at the same temperature. It is notable that the only difference between the SC-PA and the SC-KOH is the media that contains the aqueous solution of KOH; the polyampholyte network contains KOH in the SC-PA in a hydrogel form, whereas the separator that is used for the SC-KOH leaves the aqueous solution as a liquid. As mentioned in the previous discussion, the room temperature performance is shown to be comparable to the state-of-the-art values. And the electrochemical performance of the polyampholyte supercapacitor at low temperature was close to the values of other non-aqueous electrolyte supercapacitors reported at temperatures around −30 °C, without worries about electrolyte leakage^2–4^. The specific capacitance of the activated graphene based supercapacitors in ionic liquid electrolyte was ~100 F g^−1^ at a scanning rate of 1 mV s^−1^ ^2^. And specific capacitances of an ionogel-based solid-state supercapacitor were 68 and 34 F g^−1^ operating at −20 and −40 °C, respectively, at a scanning rate of 5 mV s^−1^ ^42^. To our knowledge, a specific capacitance comparable to that of our polyampholyte supercapacitor at subzero temperature has never been reported before in the literature for other aqueous-based electrolyte supercapacitor, or conventional solid-state supercapacitor using PVA-KOH gel or other gels^24^. Plus, due to the unique structure of polyampholyte networks in the hydrogel^21^, the device is still flexible at subzero temperature, which will be discussed later in this paper.

In order to understand the underlying mechanism of the improvement in supercapacitor performance at −30 °C, we performed a differential scanning calorimetry (DSC) measurement for a polyampholyte that contained the same concentration of KOH as in the SC-PA (Fig. 5c). It is well known that water molecules strongly adsorbed on hydrophilic polymer chains cannot participate in ice formation, and thus are classified as ‘non-freezable water’ that can be found unfrozen down to −190 °C^43, 44^. Likewise, strongly bound water on ionic species will form hydrated ions and will not freeze as ice, but the hydrated KOH will freeze at its eutectic temperature of −60.9 °C^45^. Based on these assumptions, we froze all ‘freezable water’ at −60.0 °C for 10 minutes, followed by bringing up the temperature to +10 °C at a slow rate of +1 °C per minute. An endothermal peak started to appear at −39.5 °C, reached a peak at −12.6 °C, and ended at −6.2 °C. The fraction of water molecules that participated in the freezing–thawing cycle was quantified by the area under the peak; here, 23.6% of water molecules were frozen at −60.0 °C. A control experiment with an aqueous solution of KOH held in place with a separator reveals that 30.4% of water molecules were frozen at the same temperature. It is unclear at the moment whether the 6.8% of the water molecules that could not be frozen due to polyampholyte chains accounted for the supercapacitor performance enhancement. The measured ionic conductivity in Fig. 5c reveals that the conductivity is a strong function of temperature, but the formation of ice is not the only dominant factor determining the change in ionic conduction. One hypothesis for the improved supercapacitor performance, however, is that the morphology of ice is connected to the performance and that the crosslinked network structure of the polyampholyte chains disrupts the crystalline growth of ice. The ‘slush ice model’ is consistent with the fact that KOH containing polyampholyte hydrogel is still flexible whereas an aqueous KOH solution is a rigid monolithic ice at −30 °C. In our parallel study^21^, we showed that the hierarchical nanostructure of polyampholyte hydrogel allows both polymer-rich (yet highly hydrated) and polymer-poor (yet populated with polymer strands) domains. Vogt *et al*. measured fast dynamics of local diffusivity of water molecules in hydrogel networks at extremely low temperatures (220 K), which shed light to the mechanism of our enhanced supercapacitor performance at low temperatures^46^. Future investigations that identify structure–property relations of polyampholyte hydrogels at low temperatures may shed light on the mechanism.

### Mechanical Flexibility and Self-Healing Properties

**Figure 6:**
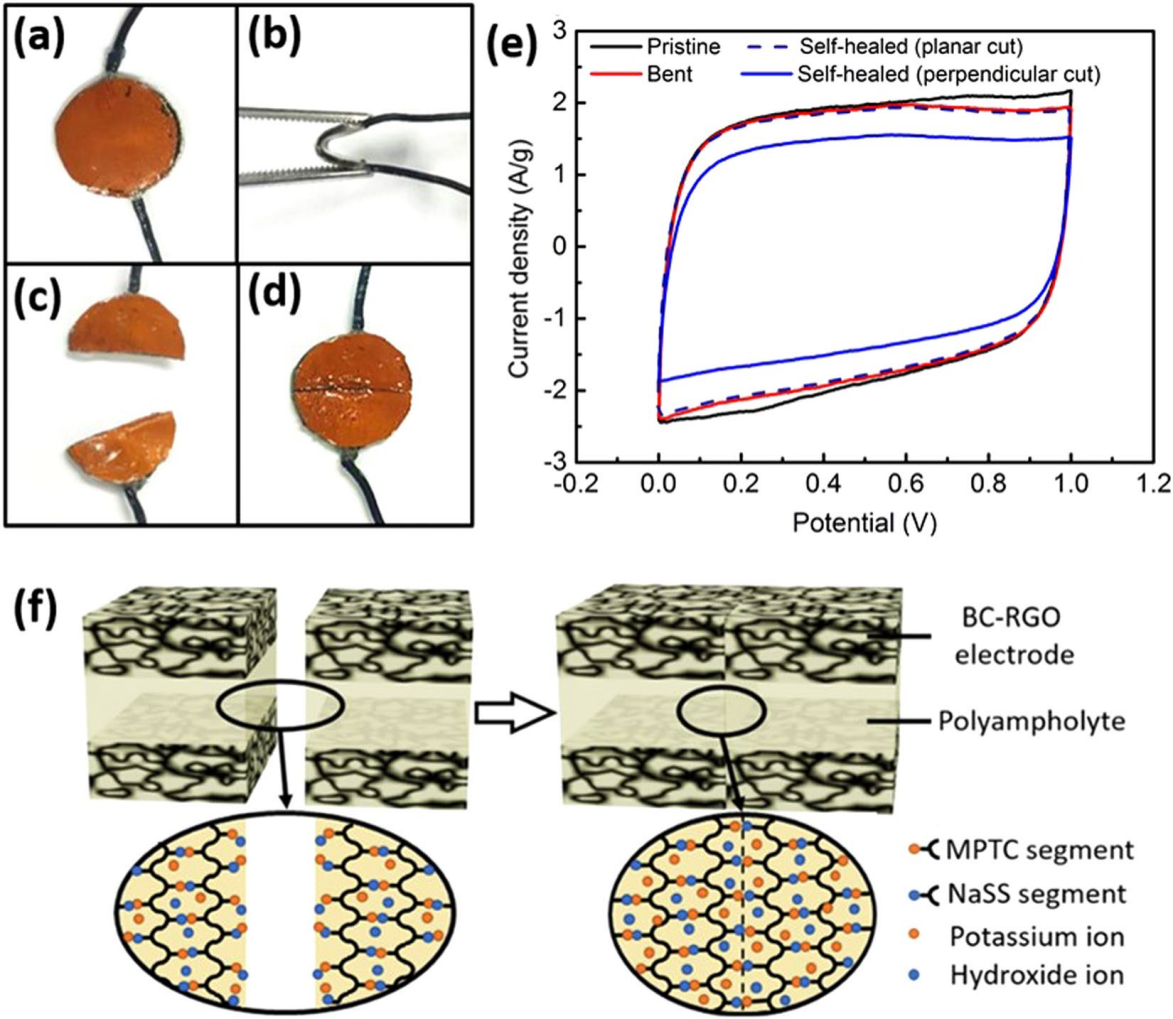
Photographs of (**a**) pristine, (**b**) bent, (**c**) broken, and (**d**) self-healed supercapacitor (SC-PA). (**c**,**d**) show the procedure for the perpendicular cut. The procedure for the planar cut is described in supporting information. (**e**) Cyclic voltammetry (CV) profile of pristine, bent and self-healed (perpendicular and planar cuts) SC-PAs at a scan rate of 20 mV s^−1^. (**f**) Schematic illustration of polyampholyte hydrogel self-healing for the perpendicular cut.

The pristine SC-PA (Fig. 6a) was bent to a radius of curvature of 5 mm (Fig. 6b), showing nearly no degradation in supercapacitor performance (Fig. 6e). The self-healing ability was tested by cutting the SC-PA into two pieces (perpendicular cut; Fig. 6c), followed by a self-healing process (Figure S9a). The self-healed SC-PA (Fig. 6d) showed ~80% of capacitance compared to the control (non-cut) device (Fig. 6e). The 20% loss of capacitance of the self-healed SC-PA may be attributed to slight mismatch in overlapping of the broken BC-electrodes, resulting in an increase of ESR. As another example of self-healing, the SC-PA was sliced into two pieces by splitting the polyampholyte hydrogel electrolyte in the planar direction (planar cut; Figure S9b). After self-healing, the CV result almost exactly reproduced the control sample result, indicating a perfect self-healing (Fig. 6e). The self-healing ability can be attributed to the reversible nature of ionic crosslinking in polyampholyte hydrogels^18, 19^, as illustrated in Fig. 6f. The bendable and self-healing nature of the SC-PA is promising for wearable and flexible electronics applications of the supercapacitor.

## Conclusions

A supercapacitor with a high energy density that works at low temperature was fabricated with a combination of BC-RGO electrodes and a polyampholyte hydrogel. Reduced graphene oxide was incorporated with biochar to transform the pyrolyzed waste biomass into high performance binder-free electrodes for electrochemical energy storage devices. The specific capacitance of BC-RGO was 216 F g^−1^ at a current density of 0.5 A g^−1^ in the three-electrode configuration. The electrochemical performance can be further improved using biochar with higher specific surface area or by doping transition metal oxide^47–49^. A symmetric supercapacitor made with BC-RGO as the electrode and polyampholyte hydrogel as the electrolyte showed a high energy density of 30 Wh kg^−1^, and a capacitance retention of ~90% after 5000 charge–discharge cycles. At low temperature (−30 °C), the SC-PA had an energy density of 10.5 Wh kg^−1^ at a power density of 500 W kg^−1^, which is a clear improvement over the performance of SC-KOH at the same temperature owing to the use of polyampholyte as a hydrogel hosting material of the aqueous KOH electrolyte, showing a potential for energy storage, even for grid-scale solutions at low temperature^50^. The flexibility and self-healing properties indicate the possible application of SC-PA in flexible and wearable devices.

## Material and Methods

### Biochar Preparation

Soybean stover was collected from agricultural fields in Korea as a model agricultural waste. The soybean stover was dried (at 60 °C), sieved (2 mm), and then pyrolyzed at 700 °C with a heating rate of 7 °C min^−1^ under anoxic conditions.

### Fabrication of BC-Treated

BC-pristine was ground and sieved to obtain fine powder (325 mesh). The powder was treated with nitric acid (35% in water) at 75 °C for 4 h, filtered, and rinsed with deionized water and ethanol^27, 28^. Drying in a convection oven at 120 °C overnight concluded the fabrication of BC-treated.

### Fabrication of BC-RGO Electrodes

BC-treated was added into a graphene oxide (GO) solution (4 mg GO mL^−1^, Graphenea, Spain). Here, 50 mg BC-treated was added to each milliliter of the GO solution. The mixture was cast on a glass plate at 80 °C for quick evaporation of the solvent. The dried BC-GO film was cut into platelet shapes 15 mm in diameter. As described in our previous paper^51^, the BC-GO platelet was reduced by an aqueous solution of L-ascorbic acid (Vitamin-C, 8 mg mL^−1^, Sigma-Aldrich, US). The reaction was done with a distillation apparatus in an oil bath at 95 °C. After 4 hours of reaction, the platelet was dialyzed in deionized water to remove remaining ascorbic acid. Then, the platelet was dried for an hour in a convection oven at 120 °C. The mass of each platelet was approximately 10 mg. The reduced platelet is denoted as BC-RGO. After that, the platelet was attached to a Kapton sheet (15 mm diameter, DuPont, US) to conclude the BC-RGO electrode fabrication.

### Synthesis of Polyampholyte Hydrogel on a BC-RGO Electrode

For polyampholyte synthesis, we followed a protocol by Gong *et al*.^18^, who developed a one-step random copolymerization of sodium 4-vinylbenzenesulfonate (cationic monomer; NaSS) and [3-(methacryloylamino)propyl] trimethylammonium chloride (anionic monomer; MPTC) with 2-hydroxy-4′-(2-hydroxyethoxy) -2-methylpropiophenone (photoinitiator). The chemicals were purchased from Sigma-Aldrich and used as received. The aqueous solution of 1 M NaSS, 1 M MPTC and 0.001 M photoinitiator was injected into the gap between two glass plates, where the gap was separated by a Teflon spacer (thickness 250 μm). Here, one of the glass plates had an attached BC-RGO electrode. A 2 hour irradiation with a UV lamp (broadband light with a maximum peak at 365 nm; Jelight, US) transforms the precursor solution into polyampholyte hydrogel. The hydrogel is denoted as PA. After the gelation, the BC-RGO/PA pair were peeled off of the glass plate, and then dialyzed in a 3 M potassium hydroxide (KOH) solution for 12 h.

### Assembly of Symmetric Supercapacitor

The symmetric supercapacitor was assembled by bonding the two BC-RGO/PA constructs by compression. The self-healing property of the PA facilitated the bonding process. The symmetric supercapacitor was dipped into a commercial room-temperature vulcanizing silicone (Shin-Etsu, Japan) for packaging. The silicone was cured for an hour to conclude the fabrication of the symmetric supercapacitor (SC-PA). A control sample of symmetric supercapacitor (SC-KOH), where the electrolyte is a 3 M aqueous solution of KOH, was also fabricated. In the control sample, the two BC-RGO electrodes are separated by a cellulose paper with macroscopic (~25 μm) open pores.

### Characterization of BC-RGO Electrodes

A field emission scanning electron microscope (FE-SEM; Zeiss Sigma) was utilized for the morphological study along with a high-resolution transmission electron microscope (HR-TEM; JEOL 2200FS). X-ray diffraction (XRD) patterns were recorded on a Rigaku RU-200B X-ray diffractometer with a rotating anode X-ray generator using Cu Kα radiation (40 kV, 110 mA). X-ray photoelectron spectroscopy (XPS) was carried out on a Kratos Axis spectrometer with monochromatized Al Kα. The C1s peak at 284.6 eV was used to calibrate all XPS spectra. A Renishaw In Via microscope system was used to collect Raman spectra from samples. A 785 nm diode laser was used as an excitation source. Surface area and porosity were investigated using nitrogen adsorption at 77 K (Autosorb-iQ, Quantachrome Instruments, US). Specific surface area was calculated from the BET adsorption isotherm and pore size distribution was calculated by NLDFT based on a slit-pore model; the calculations were done with a built-in software (ASiQwin). The electrical conductivity was evaluated with a four-point probe station (Pro4-4400, Lucas Signatone Corporation, CA, US) connected to a Keithley 2400 source measure unit.

### Electrochemical Measurements

The BC-RGO electrodes were tested in a three-electrode configuration in 3 M KOH electrolyte, where a platinum counter electrode and an Ag/AgCl reference electrode were used. Cyclic voltammetry (CV), galvanostatic charge–discharge (GCD) cycling and electrochemical impedance spectroscopy (EIS) were performed with an electrochemical station (1285 A/1260 A, Solartron, UK). The frequency range for EIS measurement was from 0.1 MHz to 10 mHz, where an open circuit potential mode with an AC perturbation of 5 mV was used. The durability of the BC-RGO electrodes was evaluated by applying 5000 successive GCD cycles at a current density of 2 A g^−1^. The specific capacitance (*C*_*s*_) of the BC-RGO electrode was calculated from the GCD curve using Equation 1:

1 $${C}_{s}=\frac{I{\rm{\Delta }}t}{m{\rm{\Delta }}V}$$

where *I* is the GCD current, Δ*t* is the discharge time, *m* is the mass of the BC-RGO electrode (excluding mass of the Kapton substrate), and Δ*V* is the potential window after the correction for IR–drop.

The symmetric supercapacitors were tested in a two-electrode configuration. The BC-RGO electrode capacitance was evaluated from the GCD curve using Equation 2:

2 $${C}_{s}=\frac{2I{\rm{\Delta }}t}{m{\rm{\Delta }}V}$$

Here, the equivalent series resistance (*ESR*) of the supercapacitor was calculated by IR-drop in the galvanostatic charge–discharge curves as in Equation 3:

3 $$ESR=\frac{{V}_{{\rm{IR}}-{\rm{drop}}}}{I}$$

Two key factors of a supercapacitor, the gravimetric energy density (*E*) and the power density (*P*), are evaluated from the discharge curves of the GCD at different current densities using Equations 4 and 5 ^52^:

4 $$E=I\int \frac{{\rm{\Delta }}Vdt}{2m}\,$$

5 $$P=\frac{E}{{\rm{\Delta }}t}$$

Conductivity values of the electrolyte at temperatures ranging from +20 °C to −30 °C were measured in the cell placed in a Peltier stage (TS102G, Instec Inc.), as described in the Supporting Information (Figure S1). Ionic conductivity is calculated according to Equation 6:

6 $$\kappa =\frac{c}{{R}_{s}}$$

where *c* is the cell constant, and *R*_*s*_ is the solution resistance at 60 KHz measured by EIS. To ensure that equilibrium was reached at a specific temperature, *R*_*s*_ values were collected after the output became stable. Likewise, supercapacitor performances (CV, GCD, and EIS) at a specific temperature were measured after 10 minutes of stabilization time.

### Differential Scanning Calorimetry (DSC) Measurements

The relative amount of freezable and non-freezable water in neat 3 M KOH solution in water and in the solution in the polyampholyte hydrogel (*i*.*e*., PA) were quantified by differential scanning calorimetry (DSC, Q1000, TA Instruments, US). The samples were tested immediately after preparation to prevent any possible loss of water through evaporation. For each measurement, 10 mg of sample was sealed in a copper sample pan. For each DSC measurement, the following thermal history was programmed: (i) temperature held at 10 °C for 10 min, (ii) cooled from +10 to −60 °C at a rate of 1 °C min^−1^, (iii) held at −60 °C for 10 min, (iv) heated to 10 °C at a rate of 1 °C min^−1^. Here, we utilized the data from the heating cycle. The frozen water content can be calculated using Equation 7 ^53^:

7 $${F}_{{\rm{frozen}},T}=\frac{{\rm{\Delta }}{H}_{{\rm{m}},T}}{{\rm{\Delta }}{H}_{{\rm{m}}}^{0}(1-C)}$$

where *F*_frozen,T_ is the fraction of frozen water at temperature *T*, ${\rm{\Delta }}{H}_{{\rm{m}}}^{0}$ is the melting enthalpy of pure water, ${\rm{\Delta }}{H}_{{\rm{m}}}^{0}$ = 333.5 J g^−1^, and *C* is the polymer concentration in polyampholyte hydrogel, which is calculated by Equation 8:

8 $$C=\frac{{m}_{{\rm{dried}}}}{{m}_{{\rm{pristine}}}}$$

where *m*_dried_ is the mass of freeze-dried hydrogel, and *m*_pristine_ is the mass of as-prepared hydrogel that contained 3 M KOH solution.

## Electronic supplementary material

Supporting Information
