# Supplementary material for: Flexible and Self-Healing Aqueous Supercapacitors for Low Temperature Applications: Polyampholyte Gel Electrolytes with Biochar Electrodes
Source: Sci Rep. 2017 May 10;7:1685. doi: 10.1038/s41598-017-01873-3 (PMC5431763; doi:10.1038/s41598-017-01873-3)
Supplement: Supplementary file 1 — Supporting Information [file 41598_2017_1873_MOESM1_ESM.pdf]

Supporting Information

Flexible and Self-Healing Aqueous Supercapacitors for Low Temperature Applications: Polyampholyte Gel Electrolytes with Biochar Electrodes

Xinda Li[a], Li Liu[a], Xianzong Wang[a], Yong Sik Ok[b], Janet A.W. Elliott[a], Scott X. Chang[c], Hyun-Joong Chung[,a]*

[a] Department of Chemical and Materials Engineering, University of Alberta, Edmonton, Alberta, T6G 1H9, Canada
[b] School of Natural Resources and Environmental Science & Korea Biochar Research Center, Kangwon National University, Chuncheon, 24341, Korea
[c] Department of Renewable Resources, University of Alberta, Edmonton, Alberta, T6G 2H1, Canada

* Corresponding Author:
E-mail: chung.hj13@ualberta.ca (H.-J. Chung), phone: +1-780-492-4790

S1) Experimental setup for low temperature measurements

Figure S1 shows photographs of the experimental setup. The written description can be found in the Experimental Section of the main text.

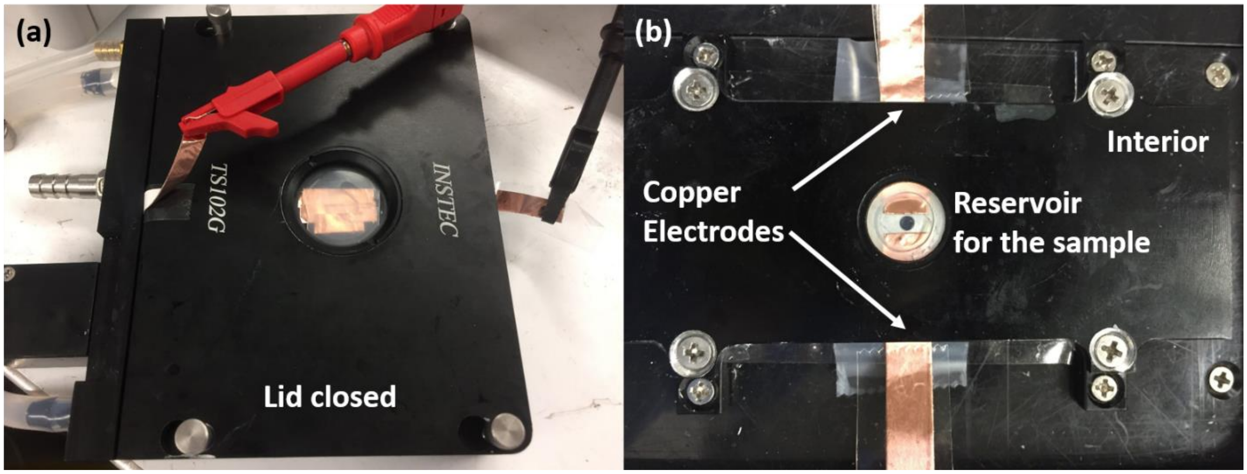

Figure S1. (a) Exterior and (b) interior pictures of our experimental setup for ionic conductivity measurement in the range between –30 to +20 °C.

S2) TEM images of BC-pristine, BC-treated and BC-RGO

For transmission electron microscope (TEM) sample preparation, 10 mg of BC-pristine (biochar at an as-received status; **Figures S2a** and **S2b**) or BC-treated (biochar after the treatment described in the Experimental Section; **Figures S2c** and **S2d**) was added to 20 mL ethanol and sonicated for 10 min. For the BC-RGO electrode (7.5 wt% RGO), the whole electrode was ground into powder before being dispersed in the ethanol. A droplet of the mixture was applied on TEM copper mesh, while any excess amount was removed with Kimwipes. The copper mesh was dried overnight in a convection oven at 120 °C.

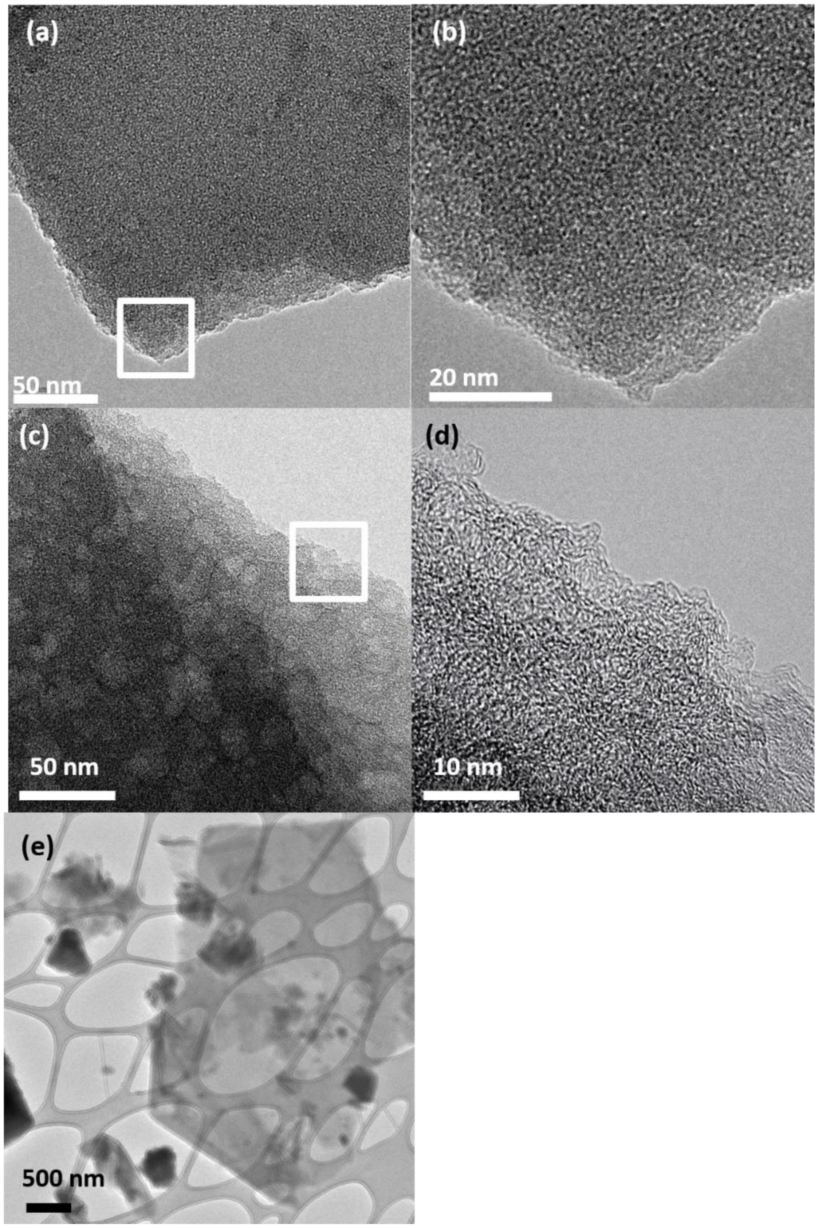

Figure S2. High resolution TEM images of BC-pristine ((a) and (b)), BC-treated ((c) and (d)), and low-resolution TEM image of BC-RGO (e). The white rectangles in the low magnification images, (a) and (c), indicate the regions of imaging in (b) and (d), respectively. See main text for discussion.

S3) Chemical analysis and electrical conductivity of biochar: Effects of treatment

Details of the measurement conditions (equipment and operation modes) can be found in the Experimental Section of the main text.

The chemical compositions at the surfaces of samples were investigated by X-Ray Photoelectron Spectroscopy (XPS). As summarized in **Table S1**, the carbon content of BC-pristine was 75.9%, with 2.4% nitrogen and 21.7% oxygen atoms. After nitric acid treatment, the oxygen and nitrogen contents were both increased to 24.0% and 4.6%, respectively. High resolution spectra of N1s (**Figure S3a**) indicates the existence of three main surface nitrogen species, pyridinic nitrogen (~398.5 eV), pyridonic nitrogen (~ 400.2 eV) and nitrate (~407.3 eV) [1, 2]. The BC-treated samples exhibited dramatically increased nitrate signal compared to the BC-pristine, possibly indicating adsorbed nitric acid after the treatment. After the reduction process by L-ascorbic acid, the peak at ~406 eV disappeared, but a strong peak of surface nitrogen oxide group was detected in the BC-RGO (the material for supercapacitor electrode; a composite of BC-treated and reduced graphene oxide). The effect of the acid treatment was further studied by analyzing C1s spectra (**Figure S3b**). The fitting suggests that the BC-treated samples have significant existence of C=O (~288.5 eV) and the amount of C-O bonding was noticeably increased as evidenced by the peak at 285.6 eV [3]. The existence of C-O bonding after the reduction by L-ascorbic acid (~285.8 eV) suggests that there are remaining hydroxyl groups on the surface of BC-RGO; we observed such a trend in our previous work [4]. It is notable that the hydroxyl groups on the surface can increase the apparent capacitance of the electrode by introducing the pseudo-capacitive effect of the quinone/hydroquinone redox pair [5, 6].

Raman spectroscopy was used to probe the bonding structure of the carbonaceous materials. There are four distinguishable bands presented in **Figure S3c**. First, the D-mode (disordered band) is located between 1330 and 1360 cm^{-1}. Second, the G-mode (tangential mode), which corresponds to the stretching mode in the graphite plane, is located at 1580 cm^{-1}. As the third and fourth bands, the second-order modes, 2D and D+G, are located at 2680–2690 and 2910–3220 cm^{-1}, respectively [8, 9]. The relative intensity of D-mode and G-mode (I_D/I_G) depends strongly on the density of defects in the graphitic material. A larger I_D/I_G suggests a higher density of sp^3 carbon due to structural defects [9]. The values of I_D/I_G decreased from 1.66 to 1.57 after the acid treatment, indicating that the structure of carbon in BC-treated is more ordered compared to that of BC-pristine, probably due to the reduction of impurities that causes the formation of amorphous carbon [10]. The intensities of 2D and D+G are also increasing from BC-pristine, to BC-treated, then to BC-RGO, indicating that the structure became more ordered as the fabrication process proceeded.

XRD patterns of BC-pristine, BC-treated, and BC-RGO are displayed in **Figure S3d**. All three samples showed two broad humps at around $2\theta = 24°$ and 43°, which are attributed to the (002) and (110) planes of graphitic carbon [11]. It is notable that BC-pristine exhibits a strong peak at $2\theta = 31°$, which belongs to a carbon allotrope with a six-fold helical chain structure whereas the bonding is entirely sp^2 hybridization [12]. Such a 6-fold peak was not observed in BC-treated and in BC-RGO, indicating a purification effect of the acid treatment.

A four-point probe measurement was used to evaluate the electrical conductivities of BC-pristine, BC-treated and BC-RGO. Here, 0.1 g of the powder of each BC was compressed into platelets at a pressure of 10 MPa. The thickness values of the platelets were obtained by cross-sectional SEM. The measured electrical conductivity values are shown in the last column of **Table S1**. The electrical conductivities of BC-pristine and BC-treated were 1.5 ± 0.2 $S{\cdot}m^{-1}$ and 0.20 ±

0.03 S·m^{-1}, respectively. After the introduction of the RGO, the conductivity value is dramatically increased to 531 ± 27 S m^{-1}. The moderately high conductivity of the powder material is very similar to the previously reported value for a KOH activated RGO electrode, which resulted in similar electrochemical performance as an electrode material for supercapacitors [17]. The scanning rate could be further increased by decreasing the electrode mass during CV [18]. This indicates that the RGO network provides an excellent conduit for electrons, and thus can serve as a charge collector in the electrochemical electrode.

Table S1. Summary of physical and chemical properties of various biochar (BC) samples.

**Samples**	S_{BET} [a] [m^{2}g^{-1}]	V_{NLDFT} [a] [cm^{3}g^{-1}]	C% [b]	N% [b]	O% [b]	I_D/I_G [c]	σ [d] [S m^{-1}]
BC-pristine	187	0.076	75.9	2.4	21.7	1.7	1.5 ± 0.2
BC-treated	414	0.26	71.4	4.6	24.0	1.6	0.20 ± 0.03
BC-RGO	483	0.35	81.5	0.8	17.7	2.0	531 ± 27

[a] Specific surface area and pore size as deduced from the adsorption isotherms in **Figure 3a**.
[b] Atomic percentage of C, O, and N as obtained from the sizes of XPS peaks.
[c] Intensity ratio between D and G bands as calculated from the Raman spectra in **Figure S3c**.
[d] Electrical conductivity values as obtained from four-point probe measurements.

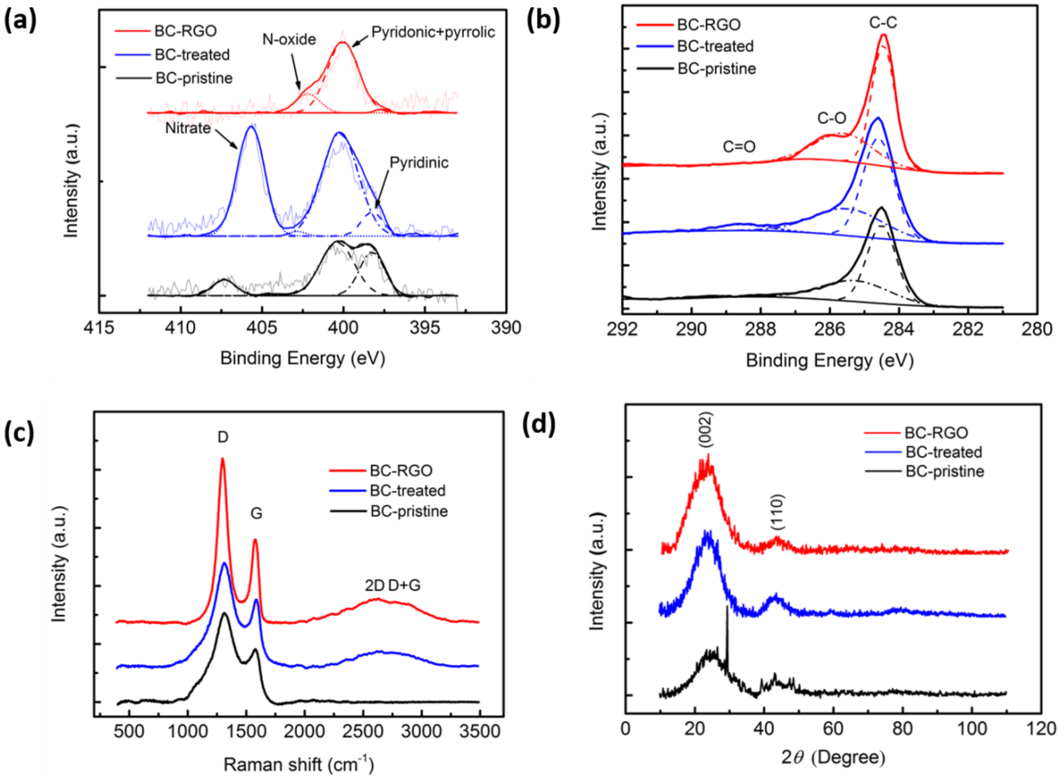

Figure S3. Chemical analysis of various biochar (BC) samples. X-ray photoelectron spectroscopy (XPS) profiles near (a) N1s and (b) C1s peaks. (c) Raman spectroscopy profiles. (d) X-ray diffraction (XRD) profiles.

S4) Electrochemical properties of BC-RGO electrodes as evaluated by the three-electrode configuration

In order to evaluate the efficacy of BC-RGO as an electrode material for supercapacitors, the three-electrode configuration measurement was performed in a 3 M KOH aqueous solution as a model electrolyte. Here, the BC-RGO was the working electrode, whereas Ag/AgCl and Pt electrodes were serving as the reference and the counter electrodes, respectively. The cyclic voltammetry (CV) results are shown in **Figure S4a.** The potential window for each voltage cycle was from –0.9 V to +0.1 V (with respect to Ag/AgCl). Various scanning rates between 5 and 100 mV s^{-1} were evaluated. No obvious redox peaks were observed in the selected potential window. **Figure S4b** gives the GCD profile of BC-RGO electrodes at varying current. The linearity in the output and charging and discharging cycle (the symmetric triangle shape of the output) implies the reversibility and stability of electrode materials during operation. The specific capacitance versus current density values are shown as the inset in **Figure S4b**; specific capacitance values were calculated from the discharging time. At current density of 0.5 A g^{-1}, the specific capacitance of the BC-RGO reached 216 F g^{-1}. The specific capacitance decreased as the current increased. One explanation is that the drop in specific capacitance of BC-RGO electrode at high current density was due to the transport behavior of ions in electrolyte when confined in the hierarchical nanostructure of electrodes. At low current density, the ions can find enough time to diffuse through the hierarchical porous structure of the BC-RGO electrodes. At high current density, the ionic transport into the nanostructures of the electrode may cause delay in redox interactions (i.e. the charge transport to the electrolyte–electrode interface becomes the rate-determining process). The rather sudden drop of specific capacitance at high current density is an issue for future study; the performance can be further enhanced by finding the right feedstock for the biochar and/or by optimizing the processing conditions for electrode fabrication. The capacitance value normalized by specific surface area was 44.62 μF cm^{-2} at 0.5 A g^{-1}. The high surface capacitance was possibly contributed to by the abundant surface functional groups, which can cause pseudo-capacitance effects. The hierarchical porous structure of BC-RGO, where macroscopic pores provide a high diffusion path for electrolytes and meso- and nanoscopic pores provide high specific surface area, is also beneficial to make the majority of pores accessible for electrochemical reactions. Moreover, the absence of binder and conductive additives enables the complete use of surface area on the surface of BC-RGO to store electrochemical energy. The Nyquist plot from an electrochemical impedance spectroscopy (EIS) measurement of BC-RGO (**Figure S4c**) comprised a semicircle in the high-frequency region and a linear plot in the low-frequency region. The equivalent series resistance (ESR) obtained by extrapolating the curve from the high-frequency region of the Nyquist plot to intersect the real-axis was only ~ 0.6 Ω. The approximately vertical line in the low-frequency region indicates fast ion transfer in aqueous electrolytes. The CV measurements of BC-RGO in various aqueous electrolyte systems were also performed (**Figure S4d**). The CV profiles show that the BC-RGO electrodes have the highest specific capacitance in 3 M KOH solution.

The results for BC-RGO electrodes with different loading amounts of RGO in the electrode were added as Figure S4e. Here, we observed that the BC-RGO provided larger specific capacitance when the electrode contained more BC-treated. The pure RGO electrodes show lower capacitance than those of BC-containing electrodes, and this can be attributed to self-aggregation or re-stacking of RGO sheets, as pointed out in previous literature [19]. Another point to consider is the mechanical integrity of the resulting BC-RGO electrode. The electrodes with RGO equal to or less than this critical value were fragile and they were occasionally broken into pieces during

the reduction in the ascorbic acid solution. Thus, even though BC-RGO electrodes with 5 wt% of RGO showed better electrochemical performance than those with 7.5 wt% RGO, the devices were fabricated with BC-RGO electrodes with 7.5 wt% RGO in the following parts of this paper.

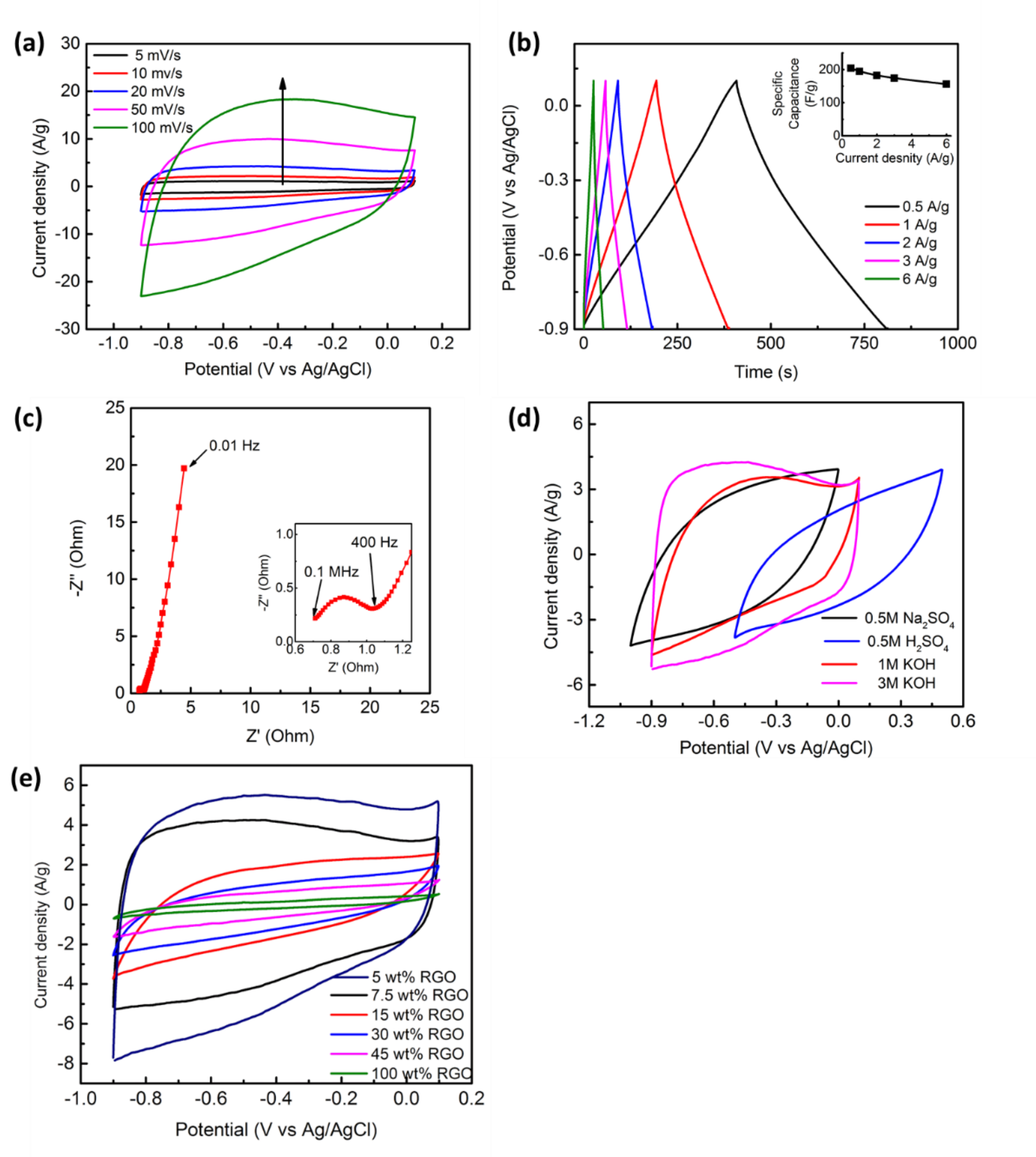

Figure S4. (a) Cyclic voltammetry (CV) profiles (b) Galvanostatic charging–discharging (GCD) profiles (inset: specific capacitance verses current density) and (c) the Nyquist plot of EIS measured in 3 M KOH aqueous electrolyte system. (d) CV of BC-RGO in different aqueous electrolytes at a scan rate of 20 mV s^{-1}. (e) CV of BC-RGO with different RGO weight percent in 3 M KOH solution at a scan rate of 20 mV s^{-1}.

S5) Device configuration of symmetric supercapacitor built with an unconfined 3 M KOH solution (SC-KOH) as the electrolyte

This sample was devised as a control sample to evaluate the efficacy of polyampholyte hydrogel as an aqueous gel electrolyte material for supercapacitors (SC-PA). Details about the fabrication and characteristics of the SC-PA control symmetric supercapacitor can be found in the main text and the following sections in the Supporting Information.

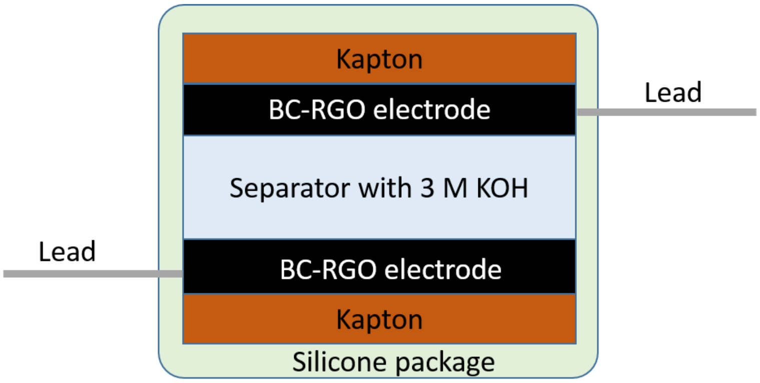

Figure S5. Device configuration of SC-KOH.

S6) Electrochemical characterizations of SC-KOH

Figures S6a and **S6b** show CV and GCD curves of SC-KOH, respectively. **Figure S6c** also compares the Nyquist plots of SC-PA and SC-KOH. ESR of SC-KOH was 0.86 Ω.

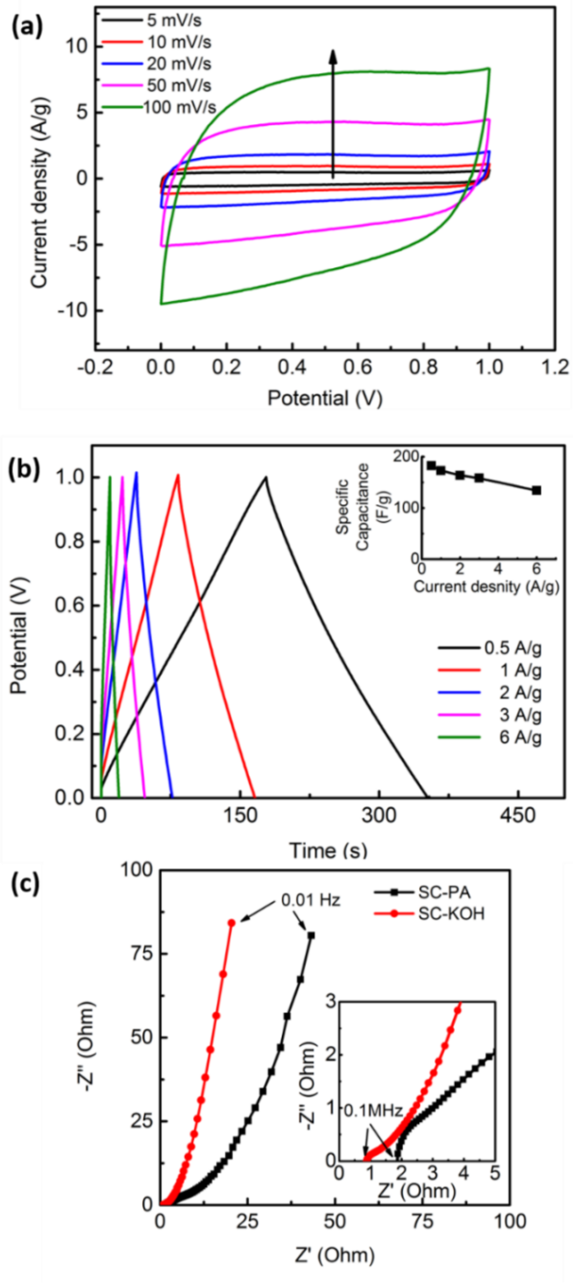

Figure S6. (a) Cyclic voltammetry (CV) profiles and (b) Galvanostatic charging–discharging (GCD) profile of SC-KOH (inset: specific capacitance verses current density). (c) Nyquist plot of EIS for SC-PA and SC-KOH. Descriptions can be found in the main text.

S7) Ragone plots of SC-PA and SC-KOH

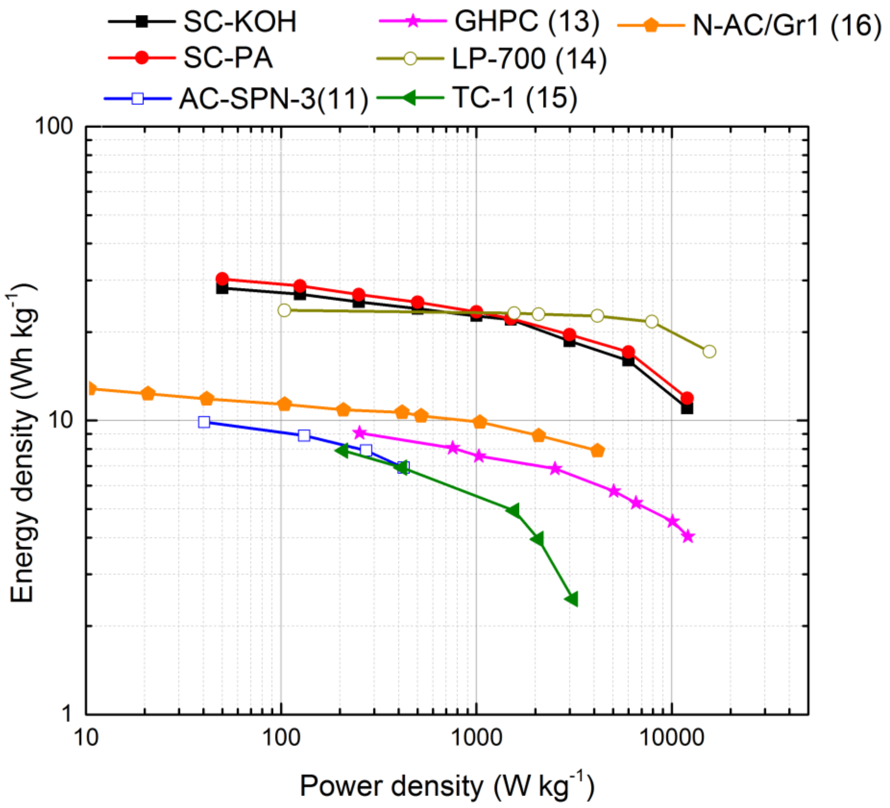

Figure S7. Performances of the SC-PA and SC-KOH devices were compared against previously published symmetric supercapacitors in references [11,13-16]. Here, the references used biomass-derived carbonaceous materials as electrodes whereas the electrolytes were in aqueous solution form.

S8) Low temperature performance of SC-KOH

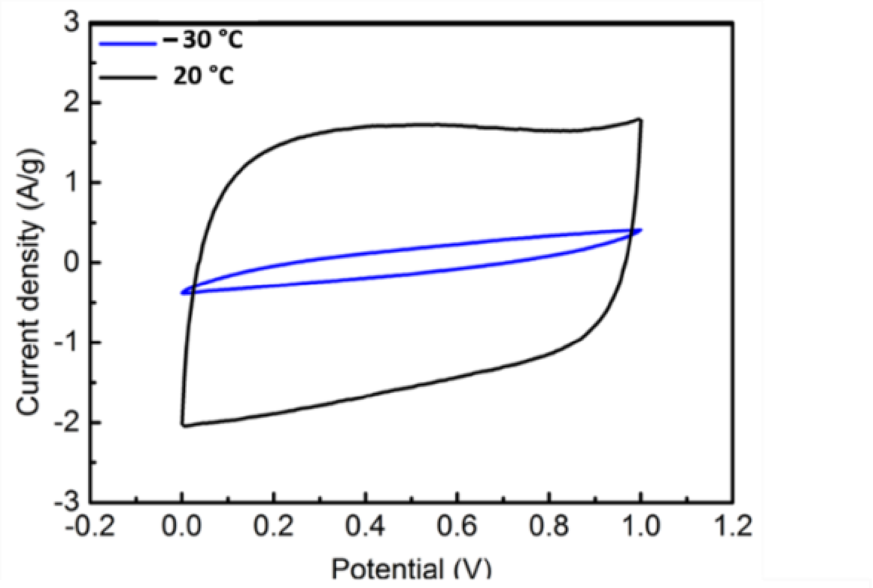

Figure S8. Cyclic voltammetry (CV) profiles of SC-KOH measured at 20 and –30 °C. The scanning rate was 20 mV s^{-1}.

S9) Flexibility and self-healing performance of SC-PA

To investigate the self-healing process for the supercapacitor, two broken parts of the SC-PA were aligned at the broken surface, followed by a mild heating and a pressure application. The setup used for SC-PA self-healing is shown in **Figure S9**. Broken SC-PA was stored in KOH solution at 30 °C. The glass slides and PTFE spacer were utilized to align the two broken pieces of the SC-PA.

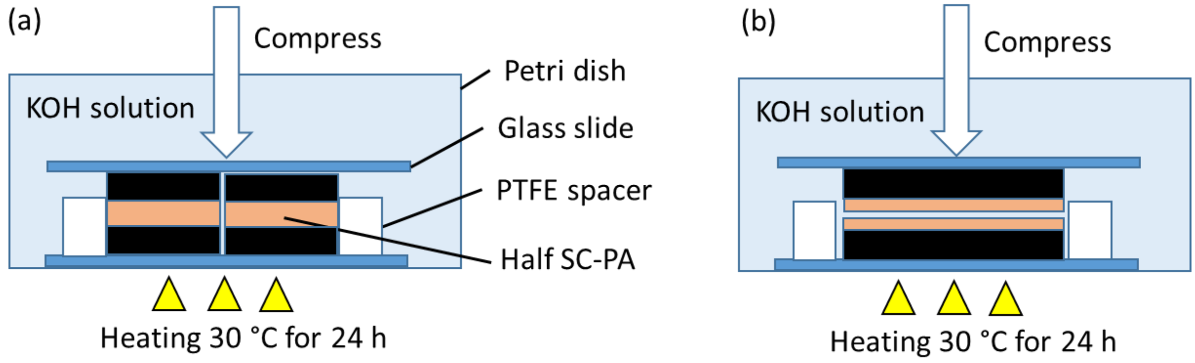

Figure S9. The experimental setup for the self-healing of SC-PA device. (a) SC-PA was cut into two pieces in the vertical direction (this describes the case for Figure 6c & d; perpendicular cut). (b) SC-PA was split into two pieces by slicing the polyampholyte hydrogel layer in the lateral direction (parallel cut).

References (for Supporting Information)

[1] B. Kumar, M. Asadi, D. Pisasale, S. Sinha-Ray, B. A. Rosen, R. Haasch, J. Abiade, A. L. Yarin, A. Salehi-Khojin, Renewable and metal-free carbon nanofibre catalysts for carbon dioxide reduction *Nat. Commun.* 4 (**2013**) 2819.
[2] J. Zhang, Z. Xia, L. Dai, Carbon-based electrocatalysts for advanced energy conversion and storage.*Sci. Adv.* 1 (**2015**) 1500564.
[3] D. R. Dreyer, S. Park, C. W. Bielawski, R. S. Ruoff, The chemistry of graphene oxide. Chem. Soc. Rev. 39 (**2010**) 228-240.
[4] X. Li, Y. Chen, A. Kumar, A. Mahmoud, J. A. Nychka, H.-J. Chung, Sponge-templated macroporous graphene network for piezoelectric ZnO nanogenerator. ACS Appl. Mater. & Interf. 7 (2015) 20753-20760.
[5] J. Jiang, L. Zhang, X. Wang, N. Holm, K. Rajagopalan, F. Chen, S. Ma, Highly ordered macroporous woody biochar with ultra-high carbon content as supercapacitor electrodes. Electrochim. Acta. 113 (2013) 481-489.
[6] G. Yu, L. Hu, N. Liu, H. Wang, M. Vosgueritchian, Y. Yang, Y. Cui, Z. Bao, Enhancing the supercapacitor performance of graphene/MnO2 nanostructured electrodes by conductive wrapping. *Nano Lett.* 11 (**2011**) 4438-4442.
[7] E. Raymundo-Piñero, F. Leroux, F. Béguin, A high-performance carbon for supercapacitors obtained by carbonization of a seaweed biopolymer. *Adv. Mater.* 18 (**2006)** 1877-1882.
[8] C. Casiraghi, A. Hartschuh, H. Qian, S. Piscanec, C. Georgi, A. Fasoli, K. Novoselov, D. Basko, A. Ferrari, Raman spectroscopy of graphene edges. *Nano Lett.* 9 (**2009)** 1433-1441.
[9] S. Stankovich, D. A. Dikin, R. D. Piner, K. A. Kohlhaas, A. Kleinhammes, Y. Jia, Y. Wu, S. T. Nguyen, R. S. Ruoff, Synthesis of graphene-based nanosheets via chemical reduction of exfoliated graphite oxide. *Carbon* 45 (**2007**) 1558-1565.
[10] M. Genovese, J. Jiang, K. Lian, N. Holm, High capacitive performance of exfoliated biochar nanosheets from biomass waste corn cob. J. Mater. Chem. A 3 (2015) 2903-2913.
[11] J. Xu, Q. Gao, Y. Zhang, Y. Tan, W. Tian, L. Zhu, L. Jiang, Preparing two-dimensional microporous carbon from Pistachio nutshell with high areal capacitance as supercapacitor materials. Sci. Rep. 4 (2014) 5545.
[12] J. T. Wang, C. Chen, E. Wang, Y. Kawazoe, A new carbon allotrope with six-fold helical chains in all-sp2 bonding networks. *Sci. Rep.* 4 (**2014**) 4339.
[13] Z. Zhu, H. Jiang, S. Guo, Q. Cheng, Y. Hu, C. Li, Dual tuning of biomass-derived hierarchical carbon nanostructures for supercapacitors: the role of balanced meso/microporosity and graphene. *Sci. Rep.* 5 (**2015**) 15936.
[14] K. L. Wang, Y. H. Cao, X. M. Wang, M. A. Castro, B. Luo, Z. R. Gu, J. Liu, J. D. Hoefelmeyer, Q. H. Fan, Rod-shape porous carbon derived from aniline modified lignin for symmetric supercapacitors *J. Power Sources* 307 (**2016**) 462-467.
[15] Y. Q. Zhao, M. Lu, P. Y. Tao, Y. J. Zhang, X. T. Gong, Z. Yang, G. Q. Zhang, H. L. Li*, Hierarchically porous and heteroatom doped carbon derived from tobacco rods for supercapacitors. J. Power Sources* 307 (**2016**) 391-400.
[16] Q. X. Xie, R. R. Bao, A. R. Zheng, Y. F. Zhang, S. H. Wu, C. Xie, P. Zhao, Sustainable low-cost green electrodes with high volumetric capacitance for aqueous symmetric supercapacitors with high energy density. *ACS Sustain. Chem. Eng.* 4 (**2016**) 1422-1430.
[17] Y. Zhu, S. Murali, M.D. Stoller, K.J. Ganesh, W. Cai, P.J. Ferreira, A. Pirkle, R.M. Wallace, K.A. Cychosz, M. Thommes, D. Su, E.A. Stach, R.A. Ruoff, Carbon-based supercapacitors produced by activation of graphene. *Science,* 332 (**2011)** 1537-1541.
[18] M. D. Stoller, R. S. Ruoff, Best practice methods for determining an electrode material's performance for ultracapacitors. *Energy Environ. Sci*. 3 **(2010)** 1294-1301.
[19] H. Huang, L. Xu, Y. Tang, S. Tang, Y. Du. Facile synthesis of nickel network supported three-dimensional graphene gel as a lightweight and binder-free electrode for high rate performance supercapacitor application. *Nanoscale* 6 **(2014)** 2426-2433.
